# Cost-effectiveness of a cardiac output-guided haemodynamic therapy algorithm in high-risk patients undergoing major gastrointestinal surgery

**DOI:** 10.1186/s13741-015-0024-x

**Published:** 2015-12-14

**Authors:** Zia Sadique, David A. Harrison, Richard Grieve, Kathryn M. Rowan, Rupert M. Pearse, for the OPTIMISE study group

**Affiliations:** Department of Health Services Research and Policy, London School of Hygiene and Tropical Medicine, London, UK; Intensive Care National Audit & Research Centre, London, UK; Queen Mary University of London, London, EC1M 6BQ UK; Adult Critical Care Unit, Royal London Hospital, Whitechapel, London, E1 1BB UK

**Keywords:** Cost-effectiveness analysis, Fluid therapy, Monitoring, CVS, Peri-operative care

## Abstract

**Background:**

The use of cardiac output monitoring to guide intra-venous fluid and inotropic therapies may improve peri-operative outcomes, but uncertainty exists regarding clinical effectiveness and robust cost-effectiveness evidence is lacking. The objective of the study was to evaluate the cost-effectiveness of peri-operative cardiac output-guided haemodynamic therapy versus usual care in high-risk patients undergoing major gastrointestinal surgery.

**Methods:**

The study undertook a cost-effectiveness analysis using data from a multi-centre randomised trial that recruited patients from 17 hospitals in the United Kingdom. The trial compared cardiac output-guided, haemodynamic therapy algorithm for intra-venous fluid and inotrope (dopexamine) infusion during and 6 h following surgery, with usual care. Resource use and outcome data on 734 high-risk trial patients aged over 50 years undergoing major gastrointestinal surgery were used to report cost-effectiveness at 6 months and to project lifetime cost-effectiveness. The cost-effectiveness analysis used information on health-related quality of life (QoL) at randomisation, 30 days, and 6 months combined with information on vital status to report quality-adjusted life years (QALYs). Each QALY was valued using the National Institute for Health and Care Excellence (NICE) recommended threshold of willingness to pay (£20,000 per QALY) in conjunction with the costs of each group to report the incremental net monetary benefits (INB) of the treatment algorithm versus usual care.

**Results:**

The mean [SD] quality of life at 30 days and 6 months was similar between the treatment groups (at 6 months, intervention group 0.73 [0.28] versus usual care group 0.71 [0.30]; mean gain 0.03 [95 % confidence interval (CI) −0.01 to 0.08]). At 6 months, survival, mean QALYs and mean healthcare costs (intervention group £8574 versus usual care group £8974) were also similar. At the cost-effectiveness threshold of £20,000 per QALY gained, the incremental net benefit of haemodynamic therapy over the patients’ lifetime was positive (£4168 [95 % CI −£3063 to £11,398]). This corresponds to an 87 % probability that this intervention is cost-effective.

**Conclusions:**

Cardiac output-guided haemodynamic therapy algorithm was associated with an average cost reduction and improvement in QALY and is likely to be cost-effective. Further research is needed to confirm the clinical and cost-effectiveness of this treatment.

**Trial registration:**

ISRCTN04386758

**Electronic supplementary material:**

The online version of this article (doi:10.1186/s13741-015-0024-x) contains supplementary material, which is available to authorized users.

## Background

More than 230 million surgical procedures are performed worldwide each year with an estimated mortality between 1 and 4 % (Pearse et al. 2012; Weiser et al. 2008). However, post-operative complications and death are much more frequent among high-risk patients, those who are older, those who have co-morbid disease and those who undergo major surgery. Importantly, patients who develop complications, but survive to leave hospital, experience a reduction in long-term survival (Pearse et al. 2011; Khuri et al. 2005). Post-operative complications increase healthcare costs significantly as a result of additional treatment and prolonged hospitals stays (Boltz et al. 2011). Implementation of new treatment strategies for this patient group therefore requires evidence of cost-effectiveness as well as clinical effectiveness.

The dose of intra-venous fluid has an important effect on patient outcomes following major gastrointestinal surgery. However, fluid is widely prescribed according to subjective criteria leading to wide variation in clinical practice (Pearse and Ackland 2012). One potential solution to this problem is the use of cardiac output monitoring to guide administration of intra-venous fluid and inotropic agents (Pearse and Ackland 2012). This approach has been recommended in a report commissioned by the Centers for Medicare and Medicaid Services in the United States of America (USA) (Agency for Healthcare Research and Quality 2007) and by the National Institute for Health and Care Excellence (NICE) in the United Kingdom (UK) (National Institute for Health and Clinical Excellence 2011). Hospitals in the UK National Health Service (NHS) receive a Commissioning for Quality and Innovation payment for each patient treated. These national policy recommendations are based on very limited health economic data. Meanwhile, the findings of a recent Cochrane review suggest that the benefit associated with this treatment may be more marginal than previously believed (Grocott et al. 2012).

In this context, the OPTIMISE trial was conducted to evaluate the clinical effectiveness of cardiac output monitoring to guide administration of intra-venous fluid and inotropic drugs as part of a haemodynamic therapy algorithm in a large, pragmatic, multi-centre and randomised controlled trial in high-risk patients undergoing major gastrointestinal surgery (Pearse et al. 2014). The aim of this analysis was to confirm whether a cardiac output-guided haemodynamic therapy algorithm is cost-effective in high-risk patients undergoing major gastrointestinal surgery.

## Methods

### Ethics, consent and permissions

The OPTIMISE trial protocol version 1.8 was approved by the East London and City Research Ethics Committee (chair: Dr A. Tucker) on 7 December 2009 (reference: 09/H0703/23) and by the Medical and Healthcare products Regulatory Agency (reference: 2009-009596-35). Written informed consent was obtained from all patients prior to inclusion in the trial.

### Setting, patients and clinical management

OPTIMISE was a pragmatic, multi-centre, randomised and observer-blinded trial conducted in 17 NHS hospitals in the UK. The trial findings, including the study protocol, are presented in detail elsewhere (Pearse et al. 2014). Adult patients, aged 50 years or over, undergoing major gastrointestinal surgery were eligible for recruitment provided they satisfied one or more predefined high-risk criteria. The intervention period commenced with induction of anaesthesia and continued until 6 hours after surgery was completed. All patients received standard measures to maintain oxygenation (SpO_2_ ≥ 94 %), haemoglobin (>8 g/dl), core temperature (37 °C), and heart rate (<100 bpm). Five percent of dextrose was administered at 1 ml/kg/h to satisfy maintenance fluid requirements with additional fluid administered at the discretion of the treating clinician guided by pulse rate, arterial pressure, urine output, core-peripheral temperature gradient, serum lactate and base excess. Mean arterial pressure was maintained between 60 and 100 mmHg using an alpha adrenoceptor agonist or vasodilator as required. Post-operative analgesia was provided by epidural infusion (bupivacaine and fentanyl) or intra-venous infusion (morphine or fentanyl). In addition, intervention group patients received intra-venous fluid and inotropic therapy guided by a haemodynamic therapy algorithm informed by cardiac output monitoring (LiDCOrapid, LiDCO Ltd, UK). This algorithm included the use of 250 ml intra-venous fluid challenges with colloid solution, as required, in order to achieve and maintain the maximal value of stroke volume. No attempt was made to standardise the choice of colloid solution. The patients in the intervention group also received an intra-venous infusion of dopexamine at a fixed rate of 0.5 μg/kg/min (Cephalon, Welwyn Garden City, UK) either through a peripheral or central venous catheter. The dose of dopexamine was reduced if the heart rate increased above 120 % of baseline value or 100 bpm (whichever was greater) for more than 30 min despite adequate anaesthesia and analgesia. If the heart rate did not decrease despite dose reduction, then the dopexamine infusion was discontinued. The patients in the control group received usual clinical care; however, the use of a dynamic central venous pressure target was recommended. Cardiac output monitoring was not used in the control group unless specifically requested by clinical staff because of patient deterioration. All other management decisions were taken by senior clinicians who retained the discretion to alter any aspect of patient care.

### Six-month outcomes

Health outcome is reported in terms of quality-adjusted life years (QALYs), which incorporate the effects of the intervention on both length of survival and quality of life. During the 6-month follow-up period, survival data were recorded by trial investigators and subsequently linked with death registrations from the Health and Social Care Information Centre (HSCIC) to give survival status and date of death for patients discharged from hospital. The patients (or their carers) were asked to complete a generic health-related quality of life questionnaire, the EuroQol (5-dimension 3-level version; EQ-5D-3L), at randomisation, 30 days and 6 months after surgery. The EQ-5D health profiles of each patient were then converted into a single summary index by applying a formula that attaches a weighting to the levels in each dimension. Patients’ EQ-5D profiles were combined with health state preference values from the UK general population (Dolan et al. 1999), to derive an EQ-5D utility index score anchored on a scale from 0 (death) to 1 (perfect health). QALYs were calculated by using the area under the curve method whereby each patient’s survival times is weighted by their corresponding quality of life scores at each time point (Manca et al. 2005). Patients who died between 1 and 6 months after randomisation were assigned zero quality of life scores at the corresponding time point. The base case analysis used the quality of life score at day 30 as the baseline measure rather than the score at randomisation, which for both arms may reflect the surgery itself.

### Healthcare resource use and costs

Resource use data were collected prospectively for each patient including information on the surgical procedure, trial intervention, length of stay in critical care and on the surgical ward. The unit costs of the surgical procedures were estimated from the NHS Payment by Results database (Department of Health 2013). These reference costs provide an average cost of both the surgery and hospital stay. To avoid double counting associated with costs of hospital stay, the costs of average length of stay and of 1 day in a post-anaesthetic recovery unit were subtracted from the national average unit cost, for each eligible surgical procedure. Critical care bed days were costed separately according to the level of care received (levels 2 and 3). No additional staff costs were considered because the intervention was specifically designed for delivery with existing peri-operative care resources. The costs of cardiac output monitoring equipment were obtained from the manufacturer (personal communication). Use of transfused blood products and intra-venous fluids was recorded for each patient and costed using unit costs provided by the NHS Blood and Transplant and the British National Formulary (Additional file 1: Table S1) (NHS Blood and Transplant 2013; Joint Formulary Committee. British National Formulary (online) London: BMJ Group and Pharmaceutical Press (accessed on 25th October 2013)). All unit costs were reported at 2012–2013 price levels.

### Six-month cost-effectiveness analysis

Cost-effectiveness analysis is based on the recognition that evidence of clinical effectiveness of interventions is necessary but not sufficient for decision making for adopting interventions. Here, it is essential to estimate the extent to which interventions represent value for money. The aim of cost-effectiveness analysis is to estimate the effect of a new treatment on the joint distribution of costs and effects. This approach can estimate the overall value of the intervention rather than accepting or rejecting value of intervention on the basis of individual tests of significance on costs and effects.

The cost-effectiveness analysis was conducted from an NHS perspective, using bivariate regression methods to correlate between costs and outcomes to report the mean (95 % confidence intervals) incremental costs and QALYs in the intervention group versus the usual care group. It needs to be highlighted that for economic analysis, the mean estimate is most required for decision-makers who will be interested in calculating the total costs of adopting an intervention and the total effects received in return for incurring these costs. The regression model was adjusted for age, gender, urgency of surgery, surgical procedure, location following surgery (critical care or standard ward), American Society of Anesthesiologists (ASA) grade, renal impairment, diabetes mellitus, risk factors for cardiac and/or respiratory disease, EQ-5D score at randomisation and included a random effect for each site. Pre-specified sub-group analyses were conducted according to urgency of surgery, surgical procedure category, and timing of patient recruitment (first ten patients recruited at each site versus all subsequent patients).

Cost-effectiveness is reported in terms of the incremental net monetary benefit (National Institute for Health and Care Excellence 2013), by valuing each incremental QALY at the relevant UK (NICE) willingness to pay threshold of £20,000 and subtracting incremental costs. A positive value of incremental net monetary benefit suggests an intervention is cost-effective. Cost-effectiveness acceptability curves were constructed by calculating the probability that the OPTIMISE intervention was cost-effective at different levels of willingness to pay for a QALY gain (from £0 to £50,000 per QALY gained) (Fenwick et al. 2004). Missing data were addressed through multiple imputation assuming that data were missing at random (Rubin 1987). Analyses were performed using STATA/IC version 13 and R version 3.0.2.

### Lifetime cost-effectiveness analysis

The long-term survival for each patient was calculated from observed survival within the first 6 months following surgery and from the predicted survival after 6 months using HSCIC data. Life expectancy after 6 months was predicted for each patient by applying age-gender adjusted excess death rates for OPTIMISE patients who survived beyond 6 months compared to the age-gender-matched UK general population (Office of National Statistics 2008). The most plausible long-term survival extrapolation was selected by comparing the relative goodness of fit and excess death rates of alternative parametric survival functions (Latimer 2013). To predict long-term quality of life, we used the mean value at 6 months for patients aged 72 years (median age of OPTIMISE patients). We predicted mean quality of life between year 1 and year 2 with a linear interpolation, such that after 2 years, the mean value for the OPTIMISE patients was similar to that of the age-matched general population. We then combined these predicted values of life expectancy and quality of life to calculate the projected lifetime QALYs. Lifetime incremental costs, incremental net monetary benefit and cost-effectiveness acceptability curves were then calculated using the same methods described for the 6-month analysis. Lifetime QALYs were discounted at 3.5 %. Results were reported over all the patients randomised and for the above pre-specified subgroups.

### Value of information analysis

Value of information analysis provides useful information about the economic value of further research into a given question. Value of information approaches require the estimation of the expected costs of continued uncertainty about the treatment decision (in this case, whether to introduce the haemodynamic algorithm) and then the calculation of the expected value of perfect information (EVPI), that is, of the evidence required to eliminate this uncertainty. EVPI is also the maximum that a decision-maker would be willing to pay for additional future research to inform this decision (Claxton 1999; Claxton and Posnett 1996). Here, the EVPI was calculated according to the cost-effectiveness results and according to the size of the eligible patient population estimated from the NHS Hospital Episodes Statistics database. We also assumed that future patients could be expected to benefit from the algorithm over 5 years and that the incremental net monetary benefit was normally distributed (Briggs et al. 2006), and we applied a 3.5 % discount rate (Claxton 1999).

### Sensitivity analyses

The following sensitivity analyses were performed to evaluate assumptions made in the primary analysis:

- Analysis including additional costs of a level 2 critical care bed day and 1 h of recovery nurse time for patients who were not transferred directly to critical care after surgery.
- Analysis including 4.5 h of additional nurse time after surgery to deliver the intervention.
- Analysis using EQ-5D values measured at randomisation as opposed to 30 days after surgery.
- Analysis estimating QALY for decedents for the time those patients were living. For decedents between randomisation and 1 month, a linear interpolation was applied between the baseline EQ-5D and the date of death when a zero EQ-5D score was applied. For decedents between 1 and 6 months where an EQ-5D score at 1 month was available, a linear interpolation was applied between the 1-month EQ-5D and the date of death when a zero EQ-5D score was applied.
- Analysis assuming quality of life remained unchanged after 6 months.
- Analysis estimating lifetime survival by applying the most plausible parametric function to the observed data for those surviving beyond 6 months following surgery, instead of assuming survival, was the same as that of the age and gender-matched general population (Latimer 2011).

## Results

### Baseline characteristics and 6-month outcomes

Of 734 patients randomised to OPTIMISE, one was randomised in error and excluded from the analysis. A further seven patients formally withdrew from the trial, and two patients were lost to follow-up. Baseline risk factors and quality of life were similar between groups (Table 1). There was no significant difference in survival at 6 months (Table 2). The mean EQ-5D-3L utility index score for survivors at month 1 and month 6, and the mean QALYs up to 6 months were similar between groups (Table 2).

**Table 1:** Baseline characteristics of patients recruited to the OPTIMISE trial

	Peri-operative cardiac output-guided haemodynamic therapy algorithm	Usual care
	(*n* = 368)	(*n* = 365)
Age (year)	71.26 (8.4)	72.20 (8.6)
Gender, male	237 (64.4)	229 (62.7)
Urgency of surgery		
Elective surgery	356 (96.7)	352 (96.4)
Non-elective surgery	12 (3.3)	13 (3.6)
Surgery†		
Upper gastrointestinal	108 (29.4)	111 (30.4)
Lower gastrointestinal	168 (45.7)	165 (45.2)
Small bowel +/− pancreas	86 (23.4)	82 (22.5)
Urological or gynaecological involving gut	5 (1.4)	4 (1.1)
No surgery performed	1 (0.3)	3 (0.8)
ASA grade^a^		
1	22 (6.0)	27 (7.4)
2	200 (54.4)	174 (47.7)
3	143 (38.9)	155 (42.5)
4	3 (0.8)	9 (2.5)
Location following surgery		
Critical care level 2	258 (70.1)	246 (67.4)
Critical care level 3	42 (11.4)	40 (11.0)
Post-anaesthetic recovery unit	10 (2.7)	9 (2.5)
General ward	58 (15.8)	70 (19.2)
Renal impairment	26 (7.1)	12 (3.3)
Diabetes mellitus	57 (15.5)	65 (17.8)
Risk factor for cardiac or respiratory disease	118 (32.1)	118 (32.3)
Baseline quality of life score^a^	0.78 (0.2)	0.77 (0.3)

Data presented as mean (SD) or *n* (%)

^a^Summaries presented after multiple imputation. †Planned surgical procedure assumed when type of surgery performed was missing for one case

**Table 2:** Mortality, EuroQol 5-dimension (EQ-5D) and quality-adjusted life years (QALY) up to 6 months for trial participants

	Peri-operative cardiac output-guided haemodynamic therapy algorithm	Usual care	Incremental effect^a^
	(*n* = 368)	(*n* = 365)	
6-month mortality	28 (7.61)	42 (11.51)	−0.40 (−0.39 to 1.14)
EQ-5D for survivors at month 1	0.66 (0.30)	0.63 (0.31)	0.02 (−0.02 to 0.06)
EQ-5D for survivors at month 6	0.73 (0.28)	0.71 (0.30)	0.03 (−0.01 to 0.08)
QALY up to 6 months	0.37 (0.11)	0.36 (0.12)	0.01 (0.00 to 0.02)

Data presented as mean (SD) or mean (95 % confidence intervals)

^a^Odds ratio for death, incremental for other estimates

### Healthcare resource use and costs

**Fig. 1:**
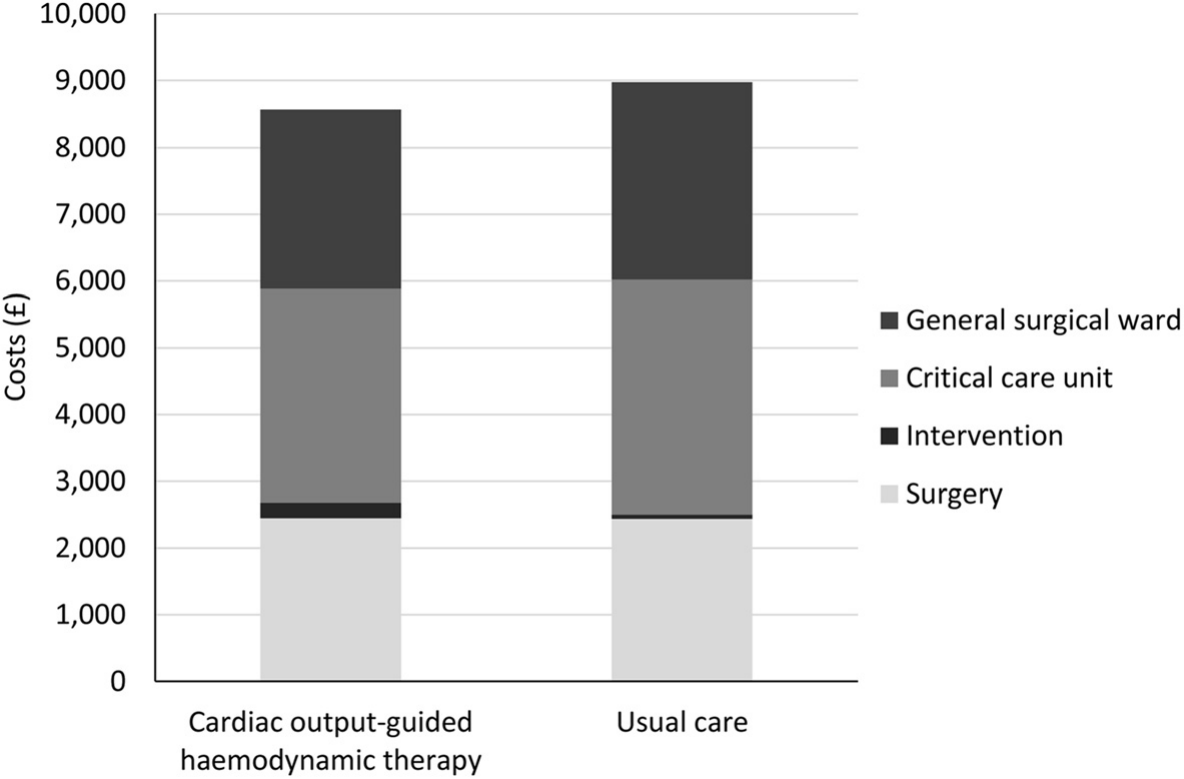
Comparison of in-hospital costs up to 6 months between peri-operative cardiac output-guided haemodynamic therapy algorithm and usual care

The mean total healthcare costs were similar in the two groups (intervention group £8574 versus usual care group £8974) (Table 3). The average cost of haemodynamic therapy during the intervention period was greater in the intervention group compared to the usual care but this cost was small compared to the total healthcare costs (intervention group £230 versus usual care group £63) (Fig. 1 and Additional file 1: Table S2). Whilst critical care utilisation and mean length of hospital stay (intervention group 13.5 days versus usual care group 14.9 days) did not differ statistically, these did contribute to the non-significant difference in total healthcare costs which more than offset the cost of the trial intervention.

**Table 3:** Hospital resource use up to 6 months for trial participants

	Peri-operative cardiac output-guided, haemodynamic therapy algorithm	Usual care
	(*n* = 368)	(*n* = 365)
Surgery		
Upper gastrointestinal	108 (29.4)	111 (30.4)
Lower gastrointestinal	168 (45.7)	165 (45.2)
Small bowel +/− pancreas	86 (23.4)	82 (22.5)
Urological or gynaecological involving gut	5 (1.4)	4 (1.1)
No surgery performed	1 (0.3)	3 (0.8)
Intervention		
Cardiac monitor used	364 (98.9)	31 (8.5)
Dopexamine dose infused (mg)	18.9 (8.4)	–
Intra-venous crystalloid (ml)		
During surgery	1518 (1410)	2420 (1382)
6 h following surgery	565 (254)	670 (367)
Intra-venous colloid (ml)		
During surgery	1465 (913)	708 (695)
6 h following surgery	642 (498)	226 (361)
Blood products (ml)		
During surgery	141 (723)	95 (542)
6 h following surgery	80 (555)	10 (66)
Length of stay (days)		
Critical care level 2	2.5 (3.7)	2.6 (3.4)
Critical care level 3	0.7 (2.4)	0.9 (3.8)
General surgical ward	10.3 (14.4)	11.4 (12.6)

Data presented as mean (SD) or *n* (%)

### Six-month cost-effectiveness analysis

At 6 months following surgery, the mean incremental costs of the intervention were negative (−£404, 95 % confidence interval (CI) −£1313 to £505), indicating that the average costs of the overall package of care were less for the trial invention versus the usual care arm (Table 4). When the QALY gain of 0.01 was valued at willingness to pay of £20,000 per QALY and the incremental cost subtracted, the incremental net monetary benefit was £580 (95 % CI −£378 to £1538) per patient (Additional file 1: Figure S1). Sub-group analyses revealed no difference in the effect of the intervention on 6-month net monetary benefits according to either urgency or type of surgery (Additional file 1: Table S3). However, in the sub-group of the later recruited patients (excluding the first ten patients enrolled at each site), the intervention was highly cost-effective, in line with the significant clinical benefit in this sub-group (Pearse et al. 2014).

**Table 4:** Hospital costs (£), quality-adjusted life years (QALY) and incremental net benefit up to 6 months and over patients’ lifetime for trial participants

	Peri-operative cardiac output-guided haemodynamic therapy	Usual care	Incremental effect
	(*n* = 368)	(*n* = 365)	
Up to 6 months			
Costs (£)	8574 (6304)	8974 (7217)	−404 (−1313 to 505)
QALY	0.37 (0.11)	0.36 (0.12)	0.01 (0.00 to 0.02)
Incremental net benefit			580 (−378 to 1538)
Lifetime			
Costs (£)	8574 (6304)	8974 (7217)	−404 (−1313 to 504)
QALY	7.59 (3.34)	7.10 (3.60)	0.19 (−0.17 to 0.54)
Incremental net benefit			4168 (−3063 to 11,398)

Data presented as mean (SD) and mean (95 % confidence intervals)

### Lifetime cost-effectiveness analysis

**Fig. 2:**
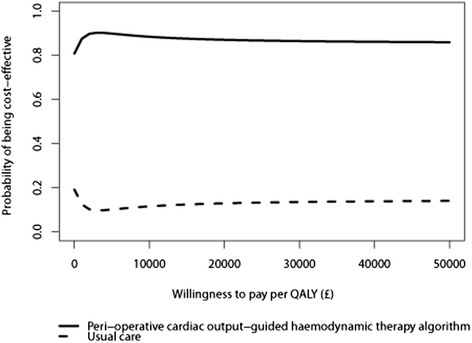
Cost-effectiveness acceptability curve (all patient lifetime analysis) describing the probability that peri-operative cardiac output-guided haemodynamic therapy is cost-effective for a range of decision makers’ willingness to pay thresholds per quality-adjusted life year (QALY) gained when compared with usual peri-operative care

The Kaplan-Meier curves suggest that, when time horizon was extended beyond 6 months for those with survival data available, the probability of survival remained similar between the two groups (Additional file 1: Figure S2). When the alternative parametric specifications were applied to the OPTIMISE data, goodness of fit (lowest AIC) was similar in the various models (Additional file 1: Table S4). Compared to the general population, each of the parametric survival models tended to predict lower mortality up to year 2 and higher mortality after year 2 (Additional file 1: Table S5). It was considered implausible that the long-term mortality estimates of high-risk surgical patients would be lower than that of the general population. In the primary analysis, we therefore applied age-gender matched general population death rates after 6 months for each comparator. Of the alternative parametric functions, the Weibull was judged the most plausible, and so, we applied this extrapolation in sensitivity analysis. The small difference in long-term quality of life between the OPTIMISE patients and the general population in the first year following surgery was assumed to decline to zero within the subsequent 2 years. The intervention group patients, experienced more average lifetime QALYs and lower costs compared with usual care patients, but again, these results were not statistically significant (Table 4). However, at the willingness to pay threshold of £20,000 per QALY recommended in the UK by NICE, the incremental net monetary benefit of the intervention versus usual care was £4168 (−£3063 to £11,398) per patient, with a corresponding 87 % probability that the intervention is cost-effective when compared with usual care (Fig. 2).

### Value of information analysis

A conservative estimate of the size of the NHS patient population eligible for the treatment was 270,503 eligible NHS patients over a 5-year period. At a threshold of £20,000 per QALY, the EVPI per patient is £239 and the total EVPI for the effective population is £65 million (Additional file 1: Figure S3).

### Sensitivity analyses

Findings of the sensitivity analyses suggested that the primary analysis was robust to alternative approaches (Additional file 1: Table S6). The incremental net monetary benefit varied between £3979 and £4289 across the scenarios studied.

## Discussion

The principal finding of this health economic evaluation was that the peri-operative, cardiac output-guided, haemodynamic therapy algorithm evaluated in the OPTIMISE trial has a high probability (87 %) of being cost-effective at the willingness to pay threshold of £20,000 per QALY gained, recommended for use in the NHS by NICE. Whereas most treatment innovations would be expected to result in a net increase in healthcare costs, lower critical care resource use and hospital stay resulted in an average cost saving amongst the intervention patients. This observation is consistent with the suggestion that improvements in the quality of peri-operative care may improve patient outcomes whilst reducing healthcare costs. The pre-specified sub-group analysis on timing of recruitment showed that the intervention was highly cost-effective in patients recruited later versus earlier at each site. This result suggests that a learning curve may have existed, consistent with expectations for trials of complex interventions. The value to the NHS of further research which would resolve the uncertainty around this treatment is £65 million, which is far greater than the likely cost of a definitive clinical trial.

The findings of this analysis are consistent with those of health economic analyses undertaken for previous similar trials. Cost-effectiveness analyses of data from two early single centre trials of peri-operative haemodynamic therapy suggested that this treatment resulted in a net reduction in healthcare costs and hence was cost-effective (Fenwick et al. 2002; Guest et al. 1997). However, more recent clinical trial data suggest that this treatment may only deliver more modest improvements in patient outcome and in some cases, provide only borderline evidence of clinical effectiveness (Grocott et al. 2012; Pearse et al. 2014). Whilst the findings of health economic simulations continue to suggest that peri-operative haemodynamic therapy may be cost saving and therefore cost-effective, these findings are sensitive to the size of treatment effect (Bartha et al. 2012; Ebm et al. 2014). Interestingly, economic evaluations of haemodynamic therapy in patients with early severe sepsis also indicate that the treatment may be cost-effective (Huang et al. 2007; Jones et al. 2011; Talmor et al. 2008). However, these analyses rely on the assumption of a strong treatment effect whilst the outcome of the recent major randomised trials suggest such protocols are associated with little or no clinical benefit in patients with severe sepsis (Peake et al. 2014; Yealy et al. 2014). Thus, whilst the findings of the current work are consistent with previous analyses, definitive evidence from a large clinical effectiveness trial would be required to confirm the economic impact of peri-operative cardiac output-guided haemodynamic therapy. Importantly, the findings of our value of information analysis show that such a trial would represent excellent value for money, even when confined to the NHS. This observation is consistent with a previously published estimate of EVPI for this treatment in elderly patients with hip fracture (Bartha et al. 2013).

OPTIMISE was the largest trial of a peri-operative, cardiac output-guided, haemodynamic therapy algorithm conducted to date, which addressed several methodological limitations in the existing evidence base (Pearse et al. 2014). The cost-effectiveness analysis was based on trial data with lifetime cost-effectiveness results extrapolated using appropriate methods and long-term survival data. Quality of life was assessed longitudinally allowing comparison of changes over time and comparison with the age-gender-matched general population. We calculated the cost of decision uncertainty along with traditional cost-effectiveness results. These findings will therefore inform both clinical policy and priorities for further research. However, costs and outcome data were only collected for 6 months and the lifetime cost-effectiveness analysis involved assumptions when extrapolating these data. Nonetheless, sensitivity analyses suggest the results were robust to alternative extrapolation methods. Some follow-up data were missing for the 6-month endpoints, which we addressed with a recommended approach of multiple imputation (Rubin 1987). Whilst health economic data were prospectively included in the trial dataset, the sample size calculation was based on clinical outcomes. These results should be interpreted in light of the fact that most trials are designed to detect differences in clinical effectiveness rather than cost-effectiveness. Healthcare costs are more variable than clinical outcomes, and economic evaluations may therefore lack statistical power. The primary objective of economic evaluation is not hypothesis testing but rather the estimation of incremental cost-effectiveness measures along with appropriate representation of the uncertainty surrounding those estimates. The results of this economic evaluation show that confidence interval of incremental costs, incremental QALY and incremental net monetary benefit included zero, implying considerable uncertainty surrounding these estimates. These uncertainties were summarised with the recommended use of cost-effectiveness acceptability curves, which conveys to decision-makers the strength of evidence in support of an intervention being cost-effective, at different levels of willingness to pay for a QALY gain (e.g. £20,000 or £30,000 per QALY).

## Conclusions

For high-risk patients undergoing major gastrointestinal surgery, the use of a peri-operative, cardiac output-guided, haemodynamic therapy algorithm was associated with an average cost reduction and is likely to be cost-effective at the willingness to pay (cost per QALY) threshold recommended for the UK by NICE. However, a further large trial is still needed to confirm both the clinical and cost-effectiveness of this treatment approach.

## Additional file

Additional file 1:
**Supplementary data describing costs and the findings of sub-group analyses and sensitivity analyses.** The additional file 1 includes supplementary information on unit costs of resources, 6 months results (hospital costs, sub-group results, cost-effectiveness acceptability curves), Kaplan-Meier survival curves and parametric extrapolation of survival beyond trial period, results of sensitivity analysis and populationexpected value of perfect information.

## Abbreviations

ASA: American Society of Anesthesiologists
EVPI: expected value of perfect information
HSCIC: Health and Social Care Information Centre
NHS: National Health Service
NICE: National Institute for Health and Care Excellence
QALY: quality-adjusted life year
UK: United Kingdom
USA: United States of America
