# Supplementary material for: Cost-effectiveness of a cardiac output-guided haemodynamic therapy algorithm in high-risk patients undergoing major gastrointestinal surgery
Source: Perioper Med (Lond). 2015 Dec 14;4:13. doi: 10.1186/s13741-015-0024-x (PMC4678585; doi:10.1186/s13741-015-0024-x)

Cost-effectiveness of a peri-operative cardiac output-guided haemodynamic therapy algorithm in high-risk patients undergoing major gastrointestinal surgery

Online Supplementary material

Z Sadique[1*], DA Harrison[2], R Grieve[1], KM Rowan[2], and RM Pearse[3] for the OPTIMISE study group

[1] Department of Health Services Research and Policy, London School of Hygiene and Tropical Medicine, London, United Kingdom

[2] Intensive Care National Audit & Research Centre, London, United Kingdom

[3] Queen Mary University of London, London, United Kingdom

Correspondence address:
Adult Critical Care Unit
Royal London Hospital
Whitechapel
London
E1 1BB
United Kingdom

Tel: +44 20 3594 0346
e-mail: r.pearse@qmul.ac.uk

Supplementary Table 1. Unit cost for individual surgical procedures.

	Items	Unit costs (£)	Source
**Monitoring**	Monitor	95	Indicative manufacturer's price
**Surgery**	Lower gastrointestinal (elective)	1,777	NHS Reference cost
	Upper gastrointestinal (elective)	2,854	NHS Reference cost
	Small bowel +/- pancreas (elective)	2,450	NHS Reference cost
	Urological or gynaecological (elective)	6,485	NHS Reference cost
	Aortic or Abdominal surgery (non-elective)	6,423	NHS Reference cost
**Blood & fluid**	Colloid (500 ml)	4.70	British National Formulary
	Crystalloid (1000 ml)	1.83	Personal communication
	Red cell (280 ml)	122.09	NHS Blood & Transplant
	Frozen fresh plasma (250 ml)	29.98	NHS Blood & Transplant
**Drug & other**	Dopexamine (50 mg)	25.20	British National Formulary
**related**	Syringe	0.36	Personal communication
	Giving set	1.53	Personal communication
	Saline	2.30	Personal communication
**Hospital costs**	Critical care level 2	868	NHS Reference cost
	Critical care level 3	1,416	NHS Reference cost
	General surgical ward	260	NHS Reference cost

Supplementary Table 2. Hospital costs (£) up to six months in the trial intervention groups.

Data presented as mean (SD).

	**Peri-operative cardiac output-guided, haemodynamic therapy algorithm (n=368)**	**Usual care (n=365)**
Surgery costs	2,453 (999)	2,440 (1019)
Intervention		
Cardiac monitor costs	94 (10)	8 (27)
Drug (Dopexamine) costs	28 (6)	-
Intravenous crystalloid costs	5 (3)	7 (3)
Intravenous colloid costs	21 (11)	9 (8)
Blood products costs	82 (379)	39 (205)
Critical care costs	3,202 (4,111)	3,519 (5,598)
General surgical ward costs	2,687 (3,751)	2,952 (3,267)
**Total costs**	**8,574 (6,304)**	**8,974 (7,217)**

Supplementary Table 3. Subgroup analyses reporting incremental costs (£), quality adjusted life years (QALYs) and incremental net benefit at six months.

Data presented as mean (95% confidence intervals). *Incremental effects are adjusted for baseline variables including all variables used in minimisation algorithm.

	**Incremental costs***	**Incremental QALY***	**Incremental net benefit***
Overall	-404 (-1,313 to 505)	0.01 (0.00 to 0.02)	580 (-378 to 1,583)
Urgency of surgery			
Elective (n=708)	-416 (-1,327 to 496)	0.01 (0.00 to 0.02)	579 (-383 to 1,541)
Non-elective (n=25)	-3,042 (-11,026 to 4,942)	-0.02 (-0.08 to 0.05)	2,733 (-5,091 to 10,557)
Type of surgery			
Upper gastro (n=222)	-273 (-2,361 to 1,815)	0.01 (-0.01 to 0.03)	454 (-1,699 to 2,607)
Lower gastro (n=334)	-796 (-1,903 to 311)	0.01 (-0.01 to 0.02)	906 (-272 to 2,083)
Small bowel +/- pancreas (n=168)	256 (-1,430 to 1,943)	0.01 (-0.01 to 0.04)	3 (-1,808 to 1,814)
Timing of recruitment			
Early (n=160)	879 (-1,257 to 3,015)	-0.02 (-0.04 to 0.01)	-1,207 (-3,442 to 1,028)
Late (n=565)	-821 (-1,840 to 197)	0.02 (0.00 to 0.03)	1,133 (61 to 2,205)

Supplementary Table 4. Goodness of fit of statistical models based on alternative distributions of survival functions.

Distribution	Akaike Information Criteria
Gompertz	1476.3
Lognormal	1509.3
Log-logistic	1482.7
Weibull	1474.6
Exponential	1474.8

Supplementary Table 5. Ratios (with 95% confidence intervals) of the mortality rates from applying alternative parametric extrapolations for OPTIMISE patients compared with the age-gender matched general population.

Year	Gompertz	Log-normal	Log-Logistic	Weibull	Exponential
**Year 1**	0.64<br>(0.55-0.72)	0.70<br>(0.62-0.78)	0.67<br>(0.59-0.75)	0.65<br>(0.57-0.73)	0.58<br>(0.51-0.65)
**Year 2**	0.98<br>(0.88-1.08)	0.96<br>(0.86-1.05)	0.97<br>(0.88-1.07)	0.97<br>(0.87-1.07)	0.98<br>(0.87-1.08)
**Year 3**	1.17<br>(1.04-1.30)	1.08<br>(0.98-1.19)	1.14<br>(1.03-1.25)	1.17<br>(1.05-1.29)	1.24<br>(1.12-1.35)
**Year 4**	1.26<br>(1.08-1.44)	1.15<br>(1.04-1.26)	1.23<br>(1.11-1.35)	1.29<br>(1.15-1.42)	1.41<br>(1.29-1.53)
**Year 5**	1.30<br>(1.08-1.52)	1.18<br>(1.06-1.29)	1.28<br>(1.15-1.40)	1.36<br>(1.22-1.50)	1.5<br>(1.39-1.63)

Supplementary Table 6. Sensitivity analyses reporting lifetime incremental costs (£), quality adjusted life years (QALYs), and incremental net benefit.

Data presented as mean (95% confidence intervals). *Incremental effects are adjusted for baseline variables including all variables used in minimisation algorithm.

	**Incremental costs***	**Incremental QALY***	**Incremental net benefit***
**Base case**	**-404 (-1,313 to 504)**	**0.19 (-0.17 to 0.54)**	**4,168 (-3,063 to 11,398)**
Total costs including costs incurred in recovery unit	-412 (-1,319 to 495)	0.19 (-0.17 to 0.54)	4,175 (-3,056 to 11,405)
Additional staff time for the intervention	-216 (-1,125 to 693)	0.19 (-0.17 to 0.54)	3,979 (-3,252 to 11,209)
Alternative QoL assumption	-405 (-1,314 to 504)	0.19 (-0.16 to 0.55)	4,289 (-3,025 to 11,603)
QALY calculation for decedents up to time of death	-405 (-1,314 to 504)	0.18 (-0.17 to 0.54)	4,097 (-3,184 to 11,379)
QoL weights from OPTIMISE	-405 (-1,314 to 504)	0.18 (-0.14 to 0.50)	4,053 (-2,470 to 10,576)
Weibull survival	-405 (-1,314 to 504)	0.19 (-0.17 to 0.54)	4,163 (-3,061 to 11,386)

Supplementary Figure 1. Cost-effectiveness acceptability curve (all patient six months analysis) describing the probability that cardiac output-guided haemodynamic therapy is cost effective for a range of decision makers' willingness to pay thresholds per quality-adjusted life year (QALY) gained when compared to usual peri-operative care.

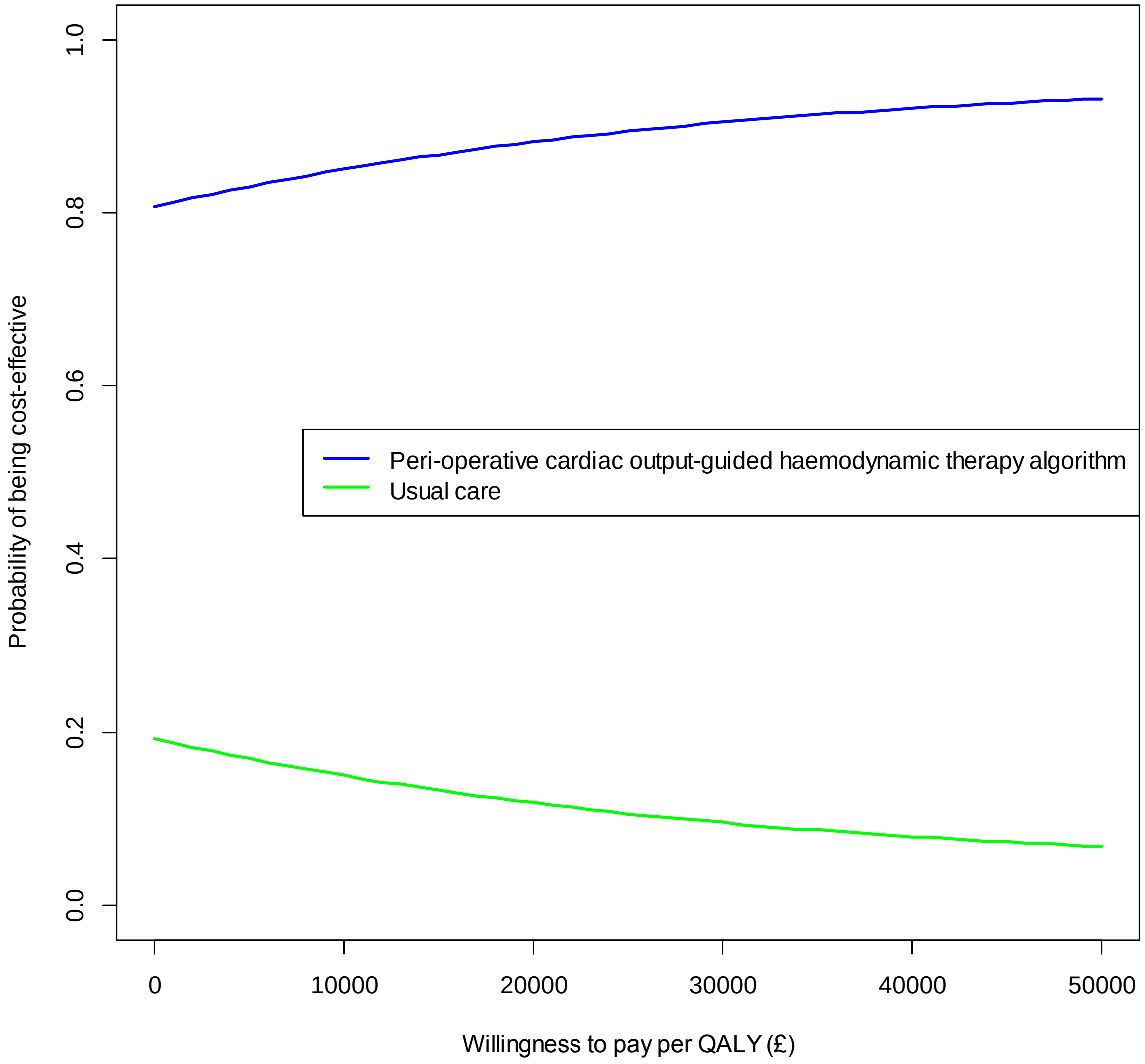

Supplementary Figure 2. Kaplan-Meier survival curves of OPTIMISE patients stratified by treatment allocation group.

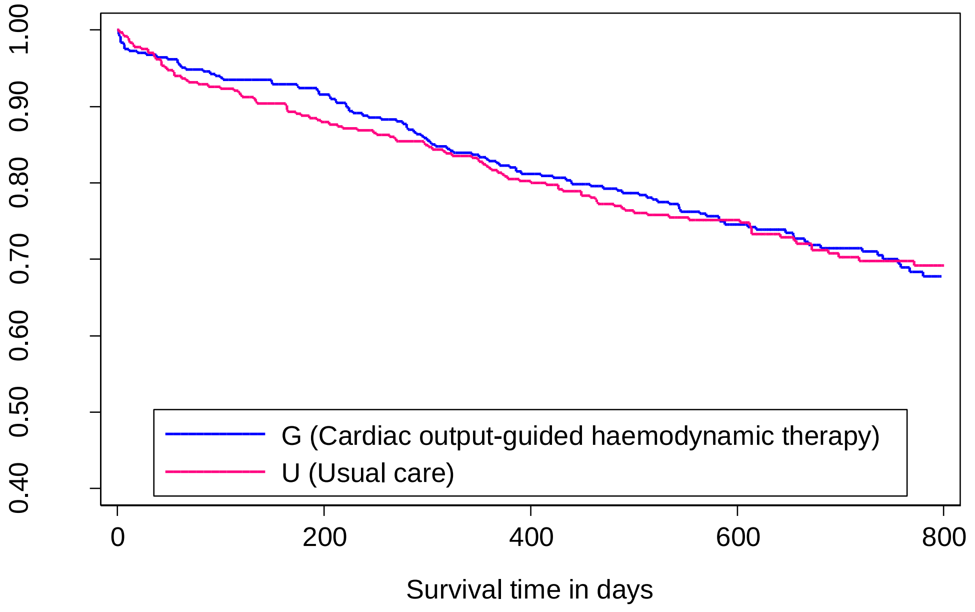

Number at risk

G	368	336	297	214	113
U	365	321	293	208	112

Supplementary Figure 3: Population expected value of perfect information (EVPI) illustrating the expected value of perfect information that would resolve uncertainty regarding the treatment decision for a range of decision makers' willingness to pay thresholds per quality-adjusted life year (QALY) gained.

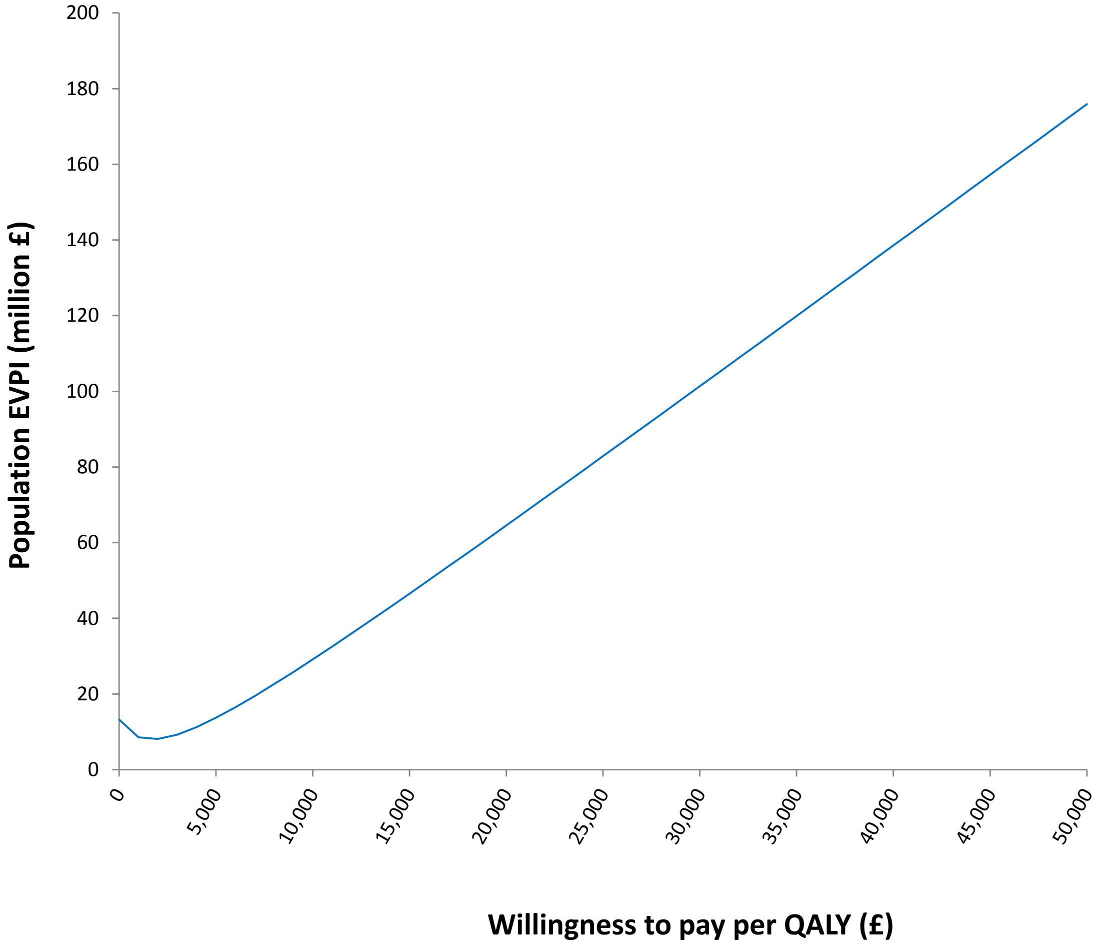

Supplement: Additional file 1: — Supplementary data describing costs and the findings of sub-group analyses and sensitivity analyses. The additional file 1 includes supplementary information on unit costs of resources, 6 months results (hospital costs, sub-group results, cost-effectiveness acceptability curves), Kaplan-Meier survival curves and parametric extrapolation of survival beyond trial period, results of sensitivity analysis and populationexpected value of perfect information. [file 13741_2015_24_MOESM1_ESM.pdf]
